# From image to insight: leveraging imaging to empower patients with inflammatory arthropathies

**DOI:** 10.3389/fmed.2025.1630114

**Published:** 2025-08-08

**Authors:** Chiara Conte, Anna Menegolo, Ludovico De Stefano, Serena Bugatti, Garifallia Sakellariou

**Affiliations:** ^1^Department of Internal Medicine and Therapeutics, University of Pavia, Pavia, Italy; ^2^Division of Rheumatology, Fondazione IRCCS Policlinico San Matteo, Pavia, Italy; ^3^Istituti Clinici Scientifici Maugeri IRCCS Pavia, Pavia, Italy

**Keywords:** rheumatoid arthritis, gout, spondyloarthritis, imaging, disability, patient education, patient engagement

## Abstract

Recent advancements in the management of inflammatory arthritis have shifted the focus toward patient-centered care, with increasing emphasis on patient education, engagement, and treatment adherence. At the same time, the growing use of imaging techniques offers novel opportunities to enhance communication between healthcare professionals and patients. This review examines current evidence on how visual tools and imaging modalities can improve patient understanding, foster engagement, and strengthen adherence in inflammatory arthritis. Barriers to adherence often arise from low health literacy and entrenched beliefs about illness and medications. However, visual communication has shown promise in addressing these challenges. Research in rheumatoid arthritis (RA), juvenile idiopathic arthritis, and gout suggest that showing personal or illustrative images can increase disease insight, reinforce treatment necessity, and encourage shared decision-making. Among imaging techniques, musculoskeletal ultrasound (MSUS) stands out for its real-time use and interactive potential. Although findings—especially in RA—are encouraging, the overall evidence remains limited, with lack of research in spondyloarthritis and scarce data on long-term outcomes. Further studies are needed to develop standardized protocols and identify patient subgroups most likely to benefit. Ultimately, integrating imaging into patient education may be a valuable strategy to improve care experiences and outcomes in inflammatory arthritis.

## 1 Introduction

Advances in the field of inflammatory arthritis, with optimal disease control becoming achievable in most patients, have shifted attention toward different aspects of care, such as comorbidities and patient-centered approaches. In this context, shared decision-making, patient engagement, and treatment adherence are gaining prominence (1), as evidence shows that involving patients in their own care leads to improved outcomes (2).

In parallel, the applications of imaging in inflammatory arthritis have expanded. Advanced techniques such as ultrasonography, magnetic resonance imaging (MRI) and computer tomography (CT) are now more accessible. Rheumatologists are also increasing able to interpret and even perform imaging, enabling multiple applications (3–5). Besides assessing disease severity and activity, imaging can also facilitate communication with patients. Given these developments, there is growing interest in integrating imaging into patient-centered care strategies for inflammatory arthritis (6).

In this narrative review, we aim to present the available evidence on the use of imaging for patient education and engagement in inflammatory arthritis. We also introduce the concept of medication adherence and explore how visual aids can be used to improve both education and patient involvement.

Rather than conducting a systematic or scoping review, we adopted a narrative approach based on our clinical expertise and a targeted literature search. We searched PubMed up to March 5, 2025, using a combination of MeSH terms and free-text keywords related to rheumatoid arthritis, psoriatic arthritis, spondyloenthesoarthritis, gout, and calcium pyrophosphate deposition disease, along with terms related to imaging modalities (ultrasound, MRI, conventional radiography, CT, scintigraphy), education, engagement, and adherence. Inclusion criteria were the application of imaging for patient education, engagement and to promote disease understanding and adherence. The full list of search terms is provided in Supplementary Table 1.

## 2 Patient involvement in the management of rheumatic diseases

Patient engagement involves equipping individuals with the tools and support needed to exert an active role in their health to improve satisfaction, outcomes, and reduce costs (7). In rheumatology, the importance of self-management, defined as ‘the ability to manage symptoms, treatment, lifestyle changes, and psychosocial and cultural consequences of health conditions’, is recognized by the European Alliance of Associations in Rheumatology (EULAR) (8). Empowering patients to develop a solid understanding of their disease is crucial for self-management and active participation in shared decision-making (8). Since self-management requires patients’ involvement and personal responsibility (9), education is considered one of its essential components (10). As disease knowledge has been positively linked with participation in healthcare (11), EULAR recommends incorporating patient education into the standard of care (12). In the context of patient-centered care, involving patients in decision-making is key to improving adherence and outcomes. However, this requires that patients have a clear understanding of treatment options, benefits, risks and their current disease status (13). Moreover, a positive healthcare practitioner-patient relationship and a clear communication are fundamental for promoting treatment adherence (14). A study on rheumatoid arthritis (RA) and ankylosing spondylitis patients found that only 25% reported high involvement, whereas the majority report some or no involvement. Importantly, higher involvement was associated with satisfaction with care (2).

### 2.1 Barriers to adherence

Medication adherence in RA, a multifaceted issue influenced by patient-related, disease-related, and drug-related factors (15), is suboptimal, often less than 50% (16). Primary non-adherence is influenced by socioeconomic factors, while secondary non-adherence is driven by lack of efficacy, slow response to treatment, and adverse reactions. Low health literacy may negatively affect both primary and secondary non-adherence (17), alongside poor social support, depression, complexity of treatments, drug costs, unsatisfactory patient-provider relationship, increased concerns about safety, and experiences of adverse effects (16, 18–21). In contrast, a consistent association with gender, disease activity, and route of administration has not been found (16).

**Figure 1:**
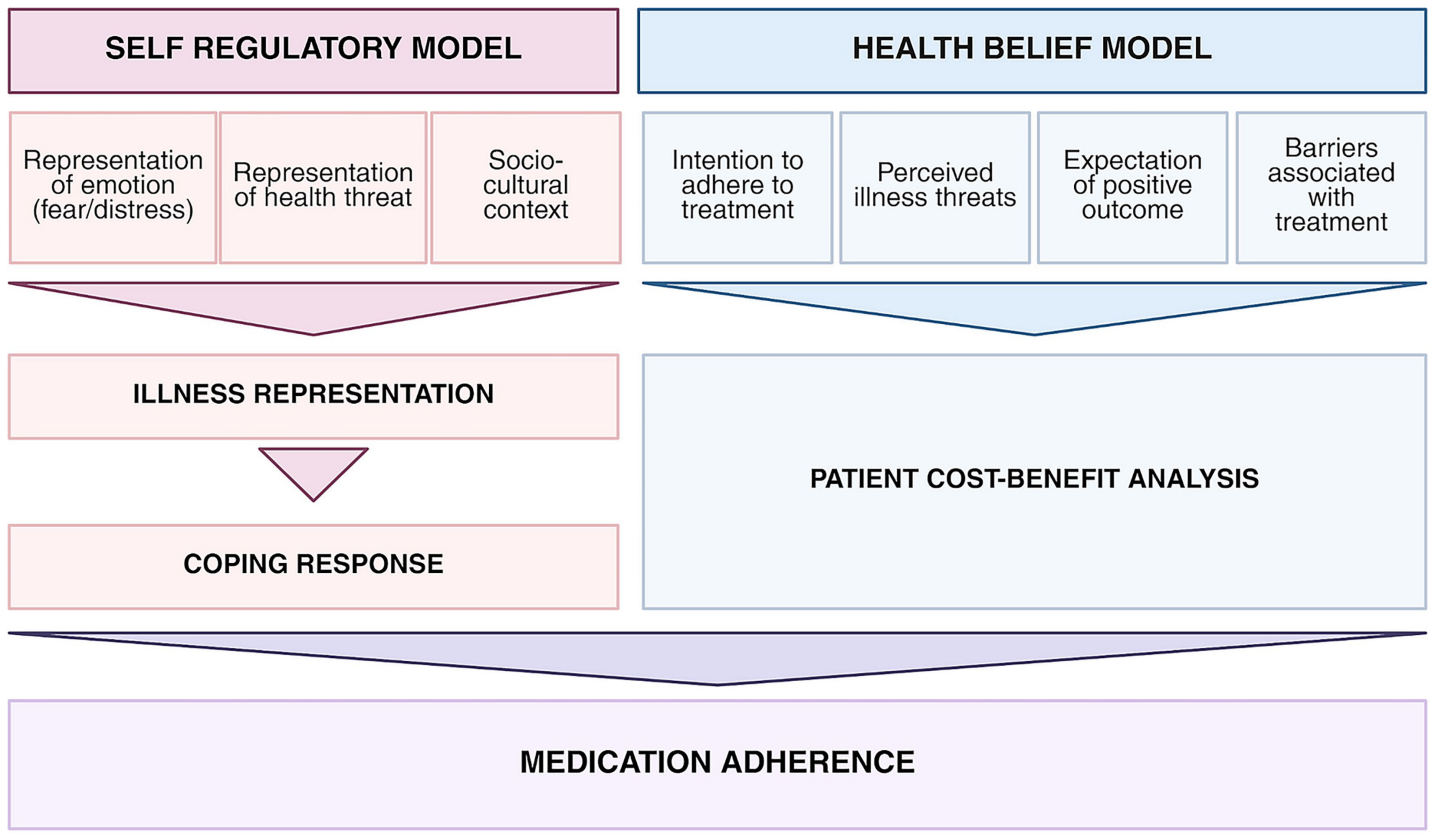
Models explaining medication adherence. https://BioRender.com/mdhc9qf.

In cases of intentional non-adherence—where a patient deliberately chooses not to follow a treatment -, the Health Belief Model provides a framework to explain this behavior. This model suggests that patient engage in an implicit cost–benefits analysis, weighting perceived risks, expectation of benefits and barriers before deciding whether to adhere (15). Another framework is the Self-Regulatory Model, for which illness representation (lay beliefs) is at the root of patients’ illness adaptation. This model suggests the influence of pre-existing beliefs on patient’s capacity to evaluate and cope with medical advice and chronic illness (15, 22). Patients who strongly believe in the necessity of a medication are more likely adhere to it (15, 23). For example, RA patients were more likely to accept treatment recommended by their rheumatologist when it aligned with their lay beliefs (24), and beliefs about medication predict early adherence to methotrexate (25), whereas non-adherence was associated with beliefs that inflammation is a natural and necessary process (26) (Figure 1).

The impact of education on adherence is not straight-forward (15), with variable success depending on the strategy. Interventions that incorporate education to daily self-management routines have shown the most promising results (16). However, a randomized trial of non-adherent RA patients found that a group-based intervention failed to change beliefs or improving adherence (27). Similarly, another trial evaluating the effect of education on adherence to sulfasalazine found no significant impact (28). Some evidence suggests that education may improve short-term outcomes, such as psychological status, depression, patient global assessment, while a correlation with long-term benefits was not identified (29). A systematic review of 7 randomized controlled trials (RCTs) on educational interventions for RA echoed these findings, highlighting short-term benefits without long-term effects (30). The focus on providing medical knowledge, without considering prior beliefs, may have limited the effect of educational interventions (24), suggesting that education alone is sometimes insufficient, as beliefs appear to be shaped by more than knowledge (16). More interactive educational interventions might result in more effective interweaving of biomedical information into pre-existing lay beliefs systems (24).

## 3 The use of images to promote patient engagement

Understanding medical information can be challenging for patients due to unfamiliar concepts and the unbalanced status between patients and health practitioners (31). Research in marketing and psychology has identify a cognitive preference for picture-based information, known as the ‘picture superiority effect’ (31). In healthcare, it has been shown that adding images to text can improve attention and stimulate patients to attend to the information (32). A randomized study of patients requiring wound repair found that those who received cartoon-based instructions were more likely to read the instructions, to answer questions correctly, and to comply with daily wound care (33). Similarly, a study about cervical cancer prevention suggested that using pictures can make educational materials more accessible (34). In rheumatology, the style of visual material can affect patient response. Illustrated booklets were more effective and photographs were the preferred format (35). Furthermore, a randomized controlled study of 111 patients with early arthritis receiving disease-modifying antirheumatic drugs (DMARDs), showed that a visual chart depicting the progression of disease activity improved outcomes compared to standard care, leading to reduction of disease activity, functional disability, quality of life, and adherence (36).

Visual aids can improve adherence (32), as shown by a randomized study of patients prescribed with antibiotics, in which pictograms in the instructions contributed to understanding and adherence (37). A Cochrane review documented that visual feedback could affect smoking cessation, diet, and healthy living (38). Showing personalized images to patients further enhance impact, as demonstrated in smokers, in which ultrasound images of carotid arteries alterations resulted in more attempts to quit (39). Overall, effectiveness of health communications can be improved by images, even though more research is needed to identify the best approach (32), and among different image styles, those deriving from imaging techniques might also be of value in this context.

### 3.1 Imaging as a support for patient engagement in inflammatory arthropathies

Visual tools to enhance patient engagement and empowerment have been explored in inflammatory arthritis, including medical imaging, defined as a range of techniques that produce visual representations of the interior of the body. Among the available imaging modalities, those offering real-time visualization, such as musculoskeletal ultrasound (MSUS), have generated particular interest. MSUS has the advantages of a ready availability in the outpatient setting, low cost, no ionizing radiations, and the capability to assess multiple sites in a single session (40). Additionally, the information can be immediately applied to patient management, supporting shared decision-making. These features make MSUS a candidate technique for patient engagement and education, with a greater impact than static imaging (15, 41).

### 3.2 Rheumatoid arthritis

Several studies have explored imaging, particularly ultrasound, for patient education and engagement in RA.

In a pilot study involving 18 patients with RA requiring treatment intensification, a single MSUS session targeting clinically affected joints was shown to increase patients’ belief in the necessity of medication. During the session, ultrasound-detected synovitis, effusion, power Doppler (PD), and bone erosions were shown and explained. Patient’s beliefs about medication, patient activation, adherence (using the Compliance Questionnaire-Rheumatology), and physical function were measured through validated questionnaires before MSUS, 3 and 10 days after. Patients’ cost–benefit analysis shifted in favor of treatment escalation after MSUS, however no changes were observed in patient activation, adherence, or disability, likely due to the small sample and the limited follow-up (25).

A qualitative study of 80 RA patients in clinical remission supported the use of MSUS to improve disease understanding and adherence. Rheumatologists performed MSUS and explained the results with each patient. Participants completed questionnaires before and after MSUS, and after 6 months. Patients found MSUS images helpful and reported an increased understanding of their disease and adherence. Rheumatologists also viewed MSUS as a valuable tool to support therapeutic decision-making (42).

MSUS can support patient education in specific groups, as shown by a study enrolling patients of South Asian origin in the UK. This group tends to show different perceptions of illness and beliefs about treatment, significant delays in seeking care, and poor adherence, contributing to worse disease outcomes.

Twenty patients were recruited, to assess if MSUS could improve disease knowledge and adherence. Patients received semi-structured interviews after reviewing three educational approaches: written leaflets, online resources to complement face-to-face interaction with health-care professionals, and MSUS during early follow-up. Patients found MSUS more useful for understanding RA and the need of medications, and it increased their motivation to participate in treatment decisions. These findings may be generalizable to other ethnic minorities or to a low-literacy background (38).

The impact of MSUS on adherence was investigated by a RCT involving 126 poorly adherent RA patients on DMARDs, with a Morisky Medication Adherence Scale (MMAS-8) < 6. Participants were randomized to receive either MSUS at baseline, or to standard care, and were followed for 6 months. Adherence was measured through self-report (MMAS-8) and pharmacy records. MSUS improved the short-term (1 month) adherence to DMARDs, but this effect was not sustained at 3 or 6 months. Additionally, this early increase in adherence did not translate into better disease control, suggesting the potential value of providing repeated MSUS feedback over time (6).

A single 12-week study explored the impact of MSUS in juvenile idiopathic arthritis (JIA). Eight patients with polyarticular or extended oligoarticular JIA and their caregivers were enrolled. A single MSUS session was performed on three or more currently or historically active joints. Both patients and caregivers found MSUS acceptable and informative, but there were no changes in adherence (measured by patient and parent questionnaires), quality of life, or disease activity (43).

Beyond MSUS, interesting results were achieved by optical spectral transmission (HandScan) of hands and wrists. In a study on 408 RA patients, the majority reported an increased insight into their disease and usefulness to monitor inflammation during medical examination (13) (Table 1).

**Table 1:** Summary of studies using imaging for patient engagement and education in rheumatoid arthritis.

Study	Population	Study design	Imaging	Outcomes	Results
Joplin 2016 (25)	18 active RA (DAS28 > 2.6) requiring treatment increase	Qualitative non-randomized study	MSUS of ≥ 1 clinically affected joint with an explanation of findings	4 questionnaires (BMQ, PAM-13, CQR, RAPID3) at baseline, 3 and 10 days post-MSUS to assess patients belief of the necessity of medication, adherence and improvement of physical function and disease activity	Showing patients MSUS resulted in an increased belief in the necessity of medication but in no change in patient activation, medication adherence or disease severity
Joshua 2019 (42)	80 RA in DAS28 remission	Qualitative non-randomized study	MSUS session with an explanation of results	Improvement in disease comprehension and adherence	All patients found MSUS useful and most thought that discussing MSUS results increased their understanding of the disease and adherence
Kumar 2020 (38)	20 RA	Qualitative non-randomized study	Three information tools: written leaflets, online information and MSUS	Change in patient perception of the disease	Patients found MSUS more useful in understanding RA and need of medications compared with other methods
Tan 2022 (6)	126 low adherent RA	RCT	MSUS	Improvement of adherence	Significantly lower rate of non-adherent patients at 1 month, but no differences at 3 and 6 months. No difference in terms of other clinical outcomes.
Favier 2018 (43)	8 adolescents with polyarticular or oligoarticular JIA and their caregivers	Qualitative non-randomized study	MSUS of three or more currently or historically active joints	Improvement of adherence, quality of life and disease activity	Both patients and caregivers found MSUS feasible, acceptable, and helpful but the intervention did not demonstrate any effect in adherence
Wolkorte 2022 (13)	408 RA	Cross-sectional	Optical spectral transmission (HandScan)	Added value to monitoring	Patients found the device useful to monitor inflammation. Respondents preferred monitoring with the device on every or most visits.
Kleyer 2017 (44)	10 healthy participants, 15 RA, 15 PsA patients	Qualitative non-randomized controlled	HR-pQCT to create 3D prototypes of MCP	Improvement of understanding of disease and adherence	3D models deeply impressed participants. 86% RA patients and 73%. PsA patients stated that they would rethink their attitude to adherence

RA, rheumatoid arthritis; DAS28, disease activity score on 28 joints; MSUS, musculoskeletal ultrasound; BMQ, Beliefs about Medication Questionnaire; PAM-13, Patient Activation Measure; CQR, Compliance Questionnaire for Rheumatology; RAPID3, Routine Assessment of Patient Index Data; RCT, randomized controlled trial; JIA, juvenile idiopathic arthritis; HR-pQCT, High-resolution peripheral quantitative computed tomography; 3D, three-dimensional; MCP, metacarpophalangeal joints; PsA, psoriatic arthritis.

### 3.3 Spondyloenthesoarthritis

Evidence on the use of imaging for patient engagement in spondyloenthesoarthritis (SpA) is very limited, with no studies assessing enthesitis and axial involvement. One study applied high-resolution peripheral quantitative computed tomography to generate 3D models of metacarpophalangeal joints in healthy individuals and patients with arthritis (15 with psoriatic arthritis (PsA) and 15 RA). The models, which clearly depicted erosions and structural damage, were shown to participants, leading to a deep impression in 66% of participants and to a change in attitude toward adherence, as revealed from qualitative interviews, in 73% of patients with PsA (44).

### 3.4 Gout and crystal-induced arthritis

The field of crystal-induced arthritis—particularly gout, a condition with poor adherence—has witnessed promising applications of imaging for patient engagement (45).

Poor clinical outcomes in gout may stem from insufficient patient education. Indeed, patients with greater disease knowledge are more likely to achieve normal serum uric acid, and participation in intensive educational sessions predicted a reduction in uric acid levels (46).

Analyses of online gout-related content have revealed a lack of effective visual communication, with 29% of the images failing to convey disease-specific information, and over half of the resources having a high level of complexity (47), highlighting the need for improvement.

In New Zealand, 204 people recruited in a supermarket—11% with gout and 44% with family or friends affected-were randomized to review one of four informational leaflets. Three leaflets provided representation of gout in the form of a cartoon, anatomical drawing, or dual-energy computed tomography scan (DECT), while the control leaflet did not contain images. Illustrated materials were found to be engaging and helped participants more efficiently in identifying treatment. While simpler illustrations conveyed information more effectively, people preferred more detailed anatomical images; DECT offered no benefit over simpler visuals (48).

Another study explored the impact of an educational intervention in 60 gout patients. Participants viewed a presentation on gout including personal DECT images, generic DECT scans, or medical illustrations from a booklet, respectively. Although no significant differences emerged between the groups, all showed increased control beliefs about the disease, increased perception of the need for treatment, improved understanding of medications and reduced perceived stigma. Participants also rated the personalized scans as the most engaging (26).

Based on the growing body of evidence, the 2023 EULAR recommendations on the use of imaging in crystal-induced arthritis state that showing and explaining imaging findings to patients may help their understanding of the disease and support adherence (40, 49).

## 4 Discussion

In this narrative review, we aimed to identify and summarize the existing evidence on the applications of imaging to support patient education and engagement in inflammatory arthritis, retrieving the relevant studies from the electronic databases.

Patient involvement has become central for managing rheumatic diseases, including inflammatory arthritis, as highlighted by its inclusion as an overarching principle in all recent EULAR recommendations (1, 50). However, the actual degree of patient involvement is only partly known and probably suboptimal, as a possible consequence of low health literacy, limited knowledge, and unbalanced status with healthcare professionals (2). Limited engagement in care can lead to reduced medication adherence, a common issue in arthritis, driven in part by patient’s beliefs (22). The importance of engagement is even more pronounced in patients that respond poorly to treatment, as recognized by the EULAR points-to-consider for the management of difficult-to-treat patients with RA (51), which include education and self-management as key components of care. In this subgroup, some patient clusters demonstrate poor adherence and limited coping strategies and which may particularly benefit from educational intervention (52).

In this context, the use of images to promote patient education, activation, and engagement emerges as a promising strategy, as also demonstrated by existing study, particularly in inflammatory arthritis (36). At the same time, imaging has become more widespread in rheumatology, with many patients undergoing multiple tests and imaging indications spread. It is increasingly common in clinical practice to show patients their personal imaging to help them understand their disease and the rationale for treatment.

Among available imaging modalities, some are more suitable for interaction, and this is particularly true for ultrasonography, which can be performed at the same time of clinical assessment, allowing a real-time explanation to the patient. For example, showing MSUS of the involved joints to patients with new-onset arthritis could improve their understanding of the disease and the necessity of the treatment. Reassessing the same joints after a course of therapy could visually demonstrate treatment response and encourage adherence. Similarly, in cases with residual pain caused by secondary sensitization without active inflammation, showing images that confirm the absence of inflammatory activity could help patients understand why the management involves pain medication rather than disease modifying drugs. Supplementary Figure 1 depicts the potential application of MSUS in patients with arthritis.

Although studies investigating the educational applications of imaging in inflammatory arthritis are limited, most have focused on MSUS in RA. These studies consistently demonstrate a positive impact of MSUS on patient’s attitudes toward medications and understanding of the disease (25, 38, 42, 43). While observational studies showed a limited effect on adherence, on RCT showed a significant-but short lived-improvement in adherence after MSUS (6). Evidence regarding other types of imaging in RA remains sparse, with one study on optical transmission imaging showing a favorable effect on disease knowledge (13). The greater use of MSUS may reflect its accessibility in rheumatology settings, with many clinicians trained to perform and interpret ultrasound directly.

In a different context, two studies investigating DECT images for education in gout (26, 48) contributed to the inclusion of a new statement in the recent EULAR recommendations on the use of imaging in crystal-induced arthritis, endorsing the use of imaging for patient engagement (40), for the first time. This reflects a broader shift toward prioritizing patient’s perspective in disease management.

From the clinician’s perspective, imaging complements and enriches the clinical assessment of inflammatory arthritis, but it is also perceived as an added value by the patients, with many expressing desire form more frequent imaging (13). Imaging might be perceived by patients as a more objective representation of disease than clinical assessment alone and therefore might be seen as more reliable. Explaining personal images to patients may deepen their insight, support shared decision-making and increase the confidence on the proposed therapeutic approach. In turn, this could lead to improved medication adherence and, indirectly, better disease outcomes (53).

The benefits of imaging for patient engagement may not be limited to joint inflammation alone but could extend to extra-articular manifestations, such as cardiovascular or pulmonary involvement. However, we failed to retrieve any study on this area, leaving this field open for future research. To translate this evidence into routine clinical practice, several barriers must be addressed. Access to imaging remains inconsistent across healthcare systems, and in some regions it is not universally available. Moreover, the time required for both acquiring and explaining images may be a limiting factor in busy clinical settings.

There are also unresolved questions surrounding the optimal application of imaging for educational purposes and patient involvement. To date, the applications have been so far limited to a few diseases (evidence in SpA, for example, is particularly limited), and all existing studies have assessed the impact of a single imaging session, thus not allowing to define the impact of repeated exposure. Additionally, standardization is lacking and it remains unclear which healthcare professional should deliver the intervention, the most appropriate candidate populations, when and how often imaging should be used. Finally, visual information needs and adequate design to be effective and not to frightening to lead to improve outcomes, and the preference for images does not automatically imply a deep understanding of their content. Strengthening the evidence base and developing standardization will expand, consequently, the use of imaging techniques to improve patient’s experience in inflammatory arthritis.
