# Supplementary material for: From image to insight: leveraging imaging to empower patients with inflammatory arthropathies
Source: Front Med (Lausanne). 2025 Aug 8;12:1630114. doi: 10.3389/fmed.2025.1630114 (PMC12370698; doi:10.3389/fmed.2025.1630114)
Supplement: Supplementary file 1 [file Supplementary_Figure_1.pdf]

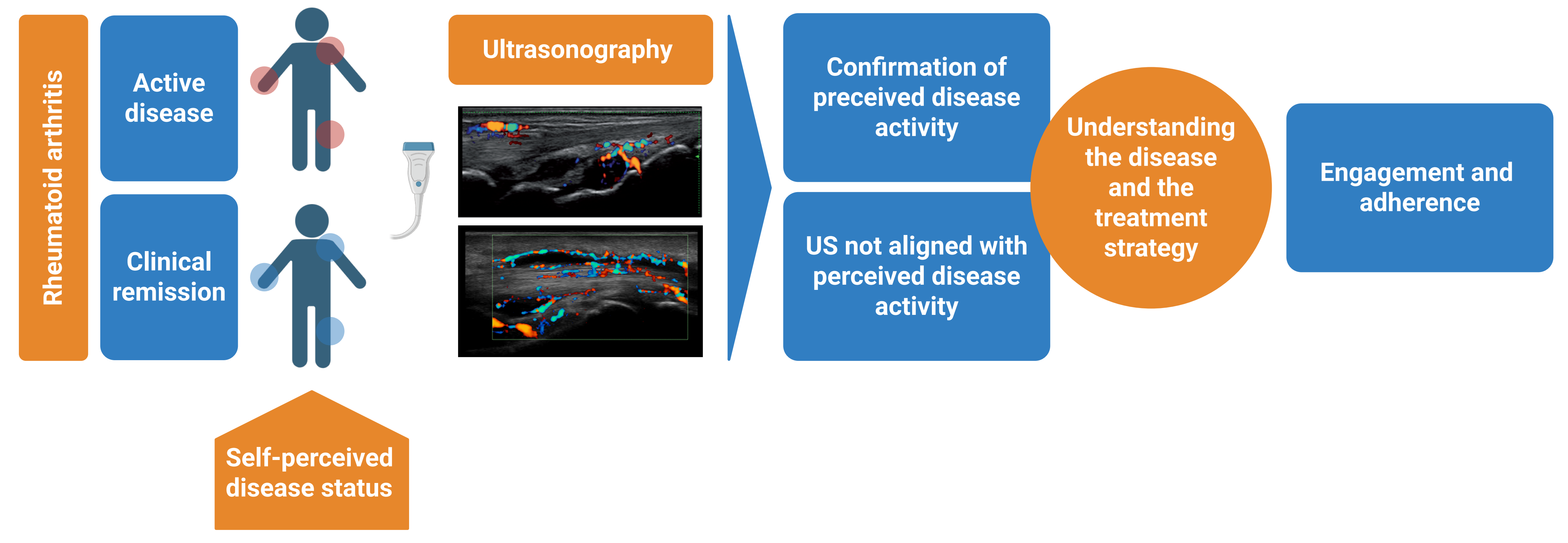

Rheumatoid arthritis
Active disease
Clinical remission
Self-perceived disease status
Ultrasonography
Confirmation of preceived disease activity
US not aligned with perceived disease activity
Understanding the disease and the treatment strategy
Engagement and adherence
