# Supplementary material for: From image to insight: leveraging imaging to empower patients with inflammatory arthropathies
Source: Front Med (Lausanne). 2025 Aug 8;12:1630114. doi: 10.3389/fmed.2025.1630114 (PMC12370698; doi:10.3389/fmed.2025.1630114)
Supplement: Supplementary file 2 [file Table_1.docx]

**From image to insight: leveraging imaging to empower patients with inflammatory arthropathies – Supplementary material**

**Supplementary Table 1: Search terms applied to PubMed**

| **Diseases** | |
| --- | --- |
| *Rheumatoid arthritis* | Arthritis, Rheumatoid [Mesh] OR “rheumatoid arthritis” OR “rheumatoid |
| *Spondyloarthritis* | Spondyloarthritis [Mesh] OR “spondyloarthr*” OR “enthes*”OR Spondylitis, Ankylosing [Mesh] OR “ankylosing spondylitis” |
| *Psoriatic arthritis* | Arthritis, Psoriatic [Mesh] OR “psoriatic arthritis” |
| *Gout* | Gout [Mesh] OR “gout” |
| *Calcium pyrophosphate deposition disease* | (“Calcium pyrophosphate” AND “arthritis”) OR “pseudogout” OR “chondrocalcinosis” |
| *Basic calcium phosphate deposition disease* | (“basic calcium phosphate” OR “hydroxyapatite”) “AND “arthritis” |
| **Imaging** | |
| *Overall* | Diagnostic Imaging [Mesh] OR “imaging” |
| *Conventional radiography* | Radiography [Mesh] OR “radiograph*” OR “X-ray*” |
| *Ultrasonography* | Ultrasonography [Mesh] OR “ultras*” |
| *Magnetic resonance imaging* | Magnetic resonance imaging [Mesh] OR “magnetic resonance imaging” OR “MRI” |
| *Computed tomography* | Tomography, X-Ray Computed [Mesh] OR “computer tomography” OR “CT” |
